# Supplementary material for: A Global Screen for Assembly State Changes of the Mitotic Proteome by SEC-SWATH-MS
Source: Cell Syst. 2020 Feb 26;10(2):133–155.e6. doi: 10.1016/j.cels.2020.01.001 (PMC7042714; doi:10.1016/j.cels.2020.01.001)

Q9Y4G8 | RPGF2_HUMAN | RAPGEF2 KIAA0313 NRAPGEP PDZGEF1
Monomer MW [kDa]: 167.417 Monomer expected elution fraction: 36

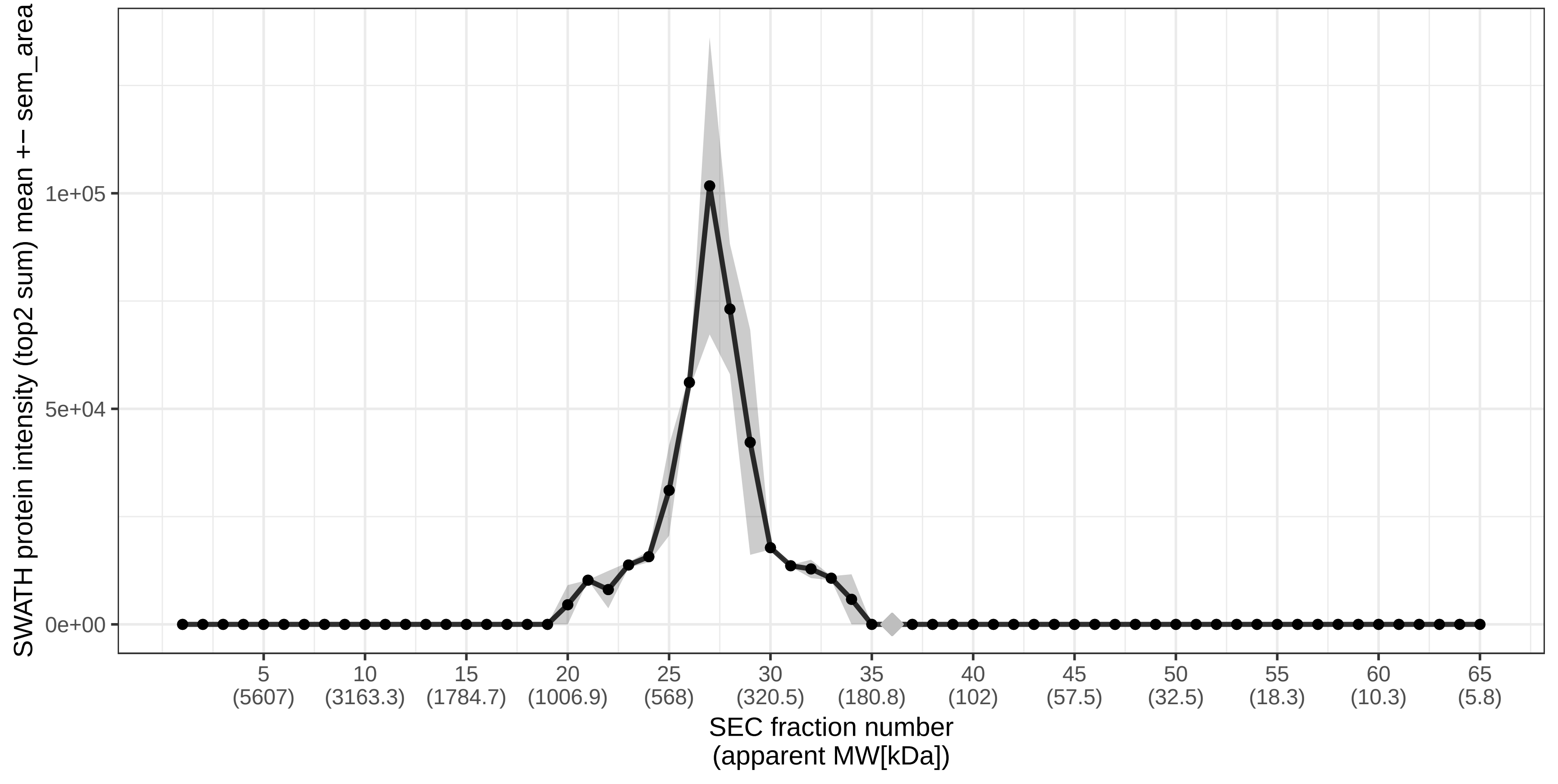

Supplement: Data S1. SEC-SWATH-MS Protein Chromatograms, Related to Figure 1 [file mmc6.zip › SECchrom_Q9Y4G8_RPGF2_HUMAN_RAPGEF2_KIAA0313_NRAPGEP_PDZGEF.pdf]
